# Exploratory strain-associated patterns of antiviral transcriptional responses to Zika virus exposure in developing human neural tissue

**DOI:** 10.1186/s44342-026-00076-5

**Published:** 2026-07-09

**Authors:** Zahra Abedi, Mohammad Ali Sheikh Beig Goharrizi, Amirreza Abbasi, Negar Sadat Soleimani Zakeri, Helia Jangi, Alireza Susanabadi Farahani

**Affiliations:** 1 Dina Pharmed Exir Salamat, Pharmaceutical Co., Tehran, 574768 Iran; 2 Department of Medical Biotechnology, School of Biotechnology, College of Science, University of Tehran, Tehran, Iran; 3 Department of Biology, Azad University of Tabriz, Tabriz, Iran; 4 Department of Software Engineering, Engineering and Architecture Faculty, Istanbul Nişantaşi University, Istanbul, Turkey; 5 Department of Biology, Ferdowsi University of Mashhad, Mashhad, Iran; 6 Department of Anesthesia and Pain, Arak University of Medical Sciences, Arak, Iran

**Keywords:** Fetal brain development, Interferon-stimulated genes, Neural progenitors, Single-cell RNA sequencing, Zika virus

## Abstract

Zika virus (ZIKV) infection affects early human brain development, but it is still not fully clear how different developmental cell lineages shape antiviral transcriptional responses. In this study, we carried out a secondary descriptive analysis using publicly available single-cell transcriptomic datasets from human fetal brain-derived neural tissue exposed ex vivo to Brazilian ZIKV-BR, the Cambodian 2010 Asian-lineage FSS13025 isolate, referred to here as ZIKV-FSS, or interferon-beta stimulation (IFNβ). Across neural lineages, radial glia-like cells enriched in progenitors and cycling NPC populations showed strong transcriptional IFN/ISG responsiveness and a higher number of genes that met exploratory differential expression criteria compared with several other cell types. Exploratory comparisons suggested strain-associated differences in response structure. In selected progenitor-focused and ISG-related summaries, the patterns linked to ZIKV-FSS showed more descriptive similarity to IFNβ-related profiles, while ZIKV-BR exposure was linked to broader and more varied gene expression changes. Sensitivity analyses showed that the transcriptional similarity between ZIKV and IFNβ depended on the gene universe used, was strongest among canonical interferon-stimulated genes (ISGs), and was more conservative across broader non-ISG and all-expressed gene sets. Contextual analyses of an independent human cerebral organoid bulk RNA-seq dataset and a Vero/moDC single-cell dataset supported the general presence of IFN/ISG-related transcriptional activation across ZIKV-exposed systems, but these were not interpreted as validation of fetal neural lineage-specific responses. Overall, these results provide an exploratory, lineage-resolved descriptive reference of antiviral transcriptional states in developing human neural tissue. Since the main fetal neural dataset included only one biological sample per condition, these findings should be interpreted as exploratory strain-associated patterns rather than definitive evidence for strain-dependent mechanisms.

**Supplementary Information:**

The online version contains supplementary material available at https://doi.org/10.1186/s44342-026-00076-5.

## Introduction

The emergence of the 2015/2016 Zika virus (ZIKV) epidemic has strongly changed global thinking about a flavivirus that was previously seen as clinically benign, and instead showed a strong causal relationship between maternal infection and severe fetal brain abnormalities, together called congenital Zika syndrome (CZS) [1, 2]. The seriousness of these findings, including microcephaly and cortical malformations, clearly highlights an unresolved basic challenge in developmental virology: why the developing human brain and its precursor cell populations are especially susceptible to ZIKV infection remains incompletely understood [3]. Although important progress has been made in understanding viral tropism and developmentally related pathologies, the transcriptional features that separate lineage-specific host responses are still not completely characterized [3, 4].

Growing evidence shows that early neural progenitor populations, including radial glia-like populations and cycling NPCs, are susceptible to ZIKV exposure and are affected during infection [3, 5, 6]. These cells have essential roles in corticogenesis by controlling progenitor expansion, neurogenesis, and cortical layering. Viral infection of these populations has been linked with cell-cycle arrest, cellular stress responses, and reduced neurogenic output [5, 6]. At the same time, several studies report strong activation of antiviral and interferon-stimulated gene (ISG) programs in neural progenitors [7, 8]. These observations create an important interpretive issue: transcriptional ISG induction in progenitor populations may represent IFN responsiveness or paracrine interferon signaling rather than cell-intrinsic antiviral initiation.

Viral strain diversity adds more complexity to this landscape. Previous experimental studies have shown that ZIKV strains can differ in neural-cell infectivity, virus production, cytotoxic effects, and induction of antiviral or innate immune-related genes [9, 10]. Although recent single-cell studies have directly profiled ZIKV-related transcriptional responses across developing human neural cell types [11], a careful secondary descriptive reanalysis can still provide a lineage-focused summary of ZIKV-associated and IFNβ-associated transcriptional patterns. In parallel, ZIKV shows a complex relationship with type I interferon (IFN-I) signaling, at the same time inducing antiviral programs while antagonizing components of IFN signaling pathways [12]. Whether ZIKV-associated transcriptional responses mainly reflect canonical IFN-I signaling or also include context-dependent differences from IFN-aligned programs remains an important question [13]. Single-cell RNA sequencing (scRNA-seq) offers a useful framework for studying these complex biological processes at cellular resolution [14]. However, secondary analyses of these public datasets need careful interpretation, especially when biological replication is limited and when comparisons are made between viral exposure conditions. Activation of antiviral responses without defining the level of convergence with IFNβ-stimulated transcriptional networks has also been reported [15]. Therefore, based on this primary atlas, a careful lineage-resolved secondary analysis remains useful for summarizing ZIKV-associated and IFNβ-associated transcriptional patterns in developing human neural tissue [11].

Beyond the main fetal neural dataset, comparisons with other ZIKV-exposed systems can give contextual information about interferon-related host responses, as long as these cross-dataset analyses are not treated as validation of neural lineage-specific findings. Recent single-cell and transcriptomic studies have shown that ZIKV-associated host responses differ across cellular contexts, including developing neural cells, dendritic cells, and non-neural reproductive tissues [11, 16, 17]. These studies suggest that ZIKV exposure can be associated with interferon-related transcriptional programs, suppression or modulation of innate immune pathways in dendritic cells, and cell-type-specific inflammatory responses in other tissues [17, 18]. In addition, host factors that control type I IFN signaling can affect ZIKV restriction [19, 20]. Together, these observations support the value of a careful, lineage-resolved descriptive analysis of ZIKV-associated and IFNβ-associated transcriptional patterns in developing human neural tissue, without assuming that observed differences between viral exposure conditions show definitive strain-dependent mechanisms.

Here, we analyze single-cell transcriptomic data from human fetal brain tissue exposed ex vivo to ZIKV-BR, the Cambodian 2010 Asian-lineage FSS13025 strain referred to here as ZIKV-FSS, or IFNβ. By comparing transcriptional responses across neural lineages and perturbations, we describe lineage-associated response levels, exploratory strain-associated transcriptional patterns, and the level of overlap between virus- and cytokine-induced programs. In addition, we put these neural responses into context using an independent human cerebral organoid bulk RNA-seq dataset and an independent ZIKV infection dataset from immune and epithelial cell types. Together, this work provides a lineage-resolved, descriptive framework for studying how developmental identity and viral exposure condition are associated with antiviral transcriptional responses during early human neurodevelopment. The contribution of this study is therefore not the creation of a new primary atlas or a mechanistic redefinition of ZIKV pathogenesis, but a careful lineage-focused secondary synthesis that standardizes cell-type-level comparisons, clearly accounts for the lack of biological replication, separates IFN/ISG responsiveness from IFNβ production or viral burden, and tests the robustness of ZIKV–IFNβ transcriptional similarity across alternative gene universes.

## Materials and methods

### Single-cell RNA-seq datasets

This study was designed as a secondary descriptive reanalysis of publicly available single-cell RNA-seq datasets. The main fetal neural dataset was taken from GSE238140, which was generated and deeply analyzed by Stokes et al. [11]. This dataset included human fetal brain-derived neural tissue exposed ex vivo to mock treatment, Brazilian ZIKV-BR, the Cambodian 2010 Asian-lineage FSS13025 isolate referred to here as ZIKV-FSS, or recombinant IFNβ. For consistency, the Brazilian ZIKV exposure condition is called ZIKV-BR, the Cambodian 2010 Asian-lineage FSS13025 exposure condition is called ZIKV-FSS, and interferon-beta stimulation is called IFNβ throughout the manuscript. Because the fetal neural dataset included one biological sample for each experimental condition, comparisons among mock, ZIKV-BR, ZIKV-FSS, and IFNβ were interpreted as descriptive and exploratory rather than as formal condition-level or strain-dependent inference. Cell numbers were not considered biological replicates. The present analysis compared cell-type-level transcriptional patterns across annotated neural lineages and exposure conditions. However, because the analysis did not separate virus-positive cells from virus-negative bystander cells and did not directly measure IFNβ production, viral RNA burden, infection frequency, or IFNAR-mediated paracrine signaling, ISG induction in progenitor populations was interpreted as transcriptional IFN/ISG responsiveness rather than evidence of cell-intrinsic innate immune initiation.

A second publicly available single-cell dataset, GSE230571, which contains ZIKV-infected Vero cells and primary human monocyte-derived dendritic cells, was used only to give a contextual comparison of interferon-associated transcriptional activation across different cellular systems [16]. This dataset was not used as independent validation of fetal neural lineage-specific findings or of radial glia/cycling NPC-specific transcriptional patterns. Each dataset was processed separately before comparative analyses. Sample metadata were downloaded from the Gene Expression Omnibus [21] and manually curated to make the annotation consistent. For the fetal neural dataset, samples were assigned to mock, ZIKV-BR, ZIKV-FSS, or IFNβ conditions based on the source metadata and sample IDs. For the Vero/moDC dataset, samples were annotated based on sample IDs and metadata that separated Vero cells from monocyte-derived dendritic cells. The curated sample metadata were added directly into Seurat objects. A complete sample mapping table, including dataset accession, GEO sample identifiers, cell system, experimental condition, analysis label, and study use, is provided in Supplementary Table 2.

### Preprocessing and quality control

Raw gene–cell count matrices were loaded with Read10X and converted into Seurat objects [22, 23]. When multi-modal 10X outputs were present, only the Gene Expression matrix was kept for downstream scRNA-seq analysis. Cells from the fetal neural dataset were kept if they met nFeature_RNA > 200, nFeature_RNA < 6000, nCount_RNA > 500, and mitochondrial transcript percentage < 12%. Cells from the Vero/moDC dataset were kept if they met nFeature_RNA > 200, nFeature_RNA < 6000, nCount_RNA > 500, and mitochondrial transcript percentage < 10%. Genes expressed in fewer than three cells were removed. After quality control, metadata fields were standardized within each dataset to make sure downstream annotation, visualization, and contextual comparison were consistent.

### Normalization, dimensionality reduction, and clustering

The fetal neural dataset was normalized using SCTransform [24], with regression of total UMI counts and mitochondrial transcript percentage. Principal component analysis (PCA) was performed, and the top 30 principal components were used to build UMAP embeddings [25] and graph-based clusters at resolution 0.4. The Vero/moDC dataset was processed separately using log-normalization, identification of 3000 variable genes, scaling, PCA, UMAP projection, and graph-based clustering at resolution 0.3. Diagnostic plots, including elbow plots, were checked to confirm that the selected principal components captured the main sources of transcriptional variation used for downstream visualization and descriptive analyses.

### Cell-type annotation

Cellular identities were assigned using canonical markers for main cortical and glial lineages [26, 27]. The radial glia cluster was identified by expression of SOX2, PAX6, and NES. Because these markers are shared across several neural progenitor states, including radial glia-like and intermediate progenitor-like populations, this cluster was interpreted as a progenitor-enriched radial glia-like population rather than a fully separated radial glia subtype. We did not further divide this cluster into apical radial glia, outer/basal radial glia, or intermediate progenitor subtypes, and therefore findings for this cluster were interpreted as progenitor-enriched radial glia-like transcriptional patterns rather than subtype-specific radial glia responses. Cycling NPCs were annotated using MKI67, TOP2A, and HMGB2; excitatory neurons using SLC17A7 and NEUROD6; inhibitory neurons using GAD1, GAD2, and DLX1; astrocytes using GFAP and AQP4; microglia using C1QA, C1QB, and TYROBP; and OPCs using PDGFRA, OLIG1, and OLIG2. Marker distributions were visualized with dot plots and compared with unsupervised clusters to assign cell-type identities. Annotated UMAPs were used as the structural framework for downstream descriptive analyses. The marker genes used for cell-type annotation and the curated ISG panel used for signature analyses are listed in Supplementary Table 33.

### Pseudobulk construction

To summarize expression while keeping sample and cell-type labels, expression values were collapsed by sample and annotated cell type using AggregateExpression in Seurat [22, 23]. This produced a pseudobulk expression matrix for visualization and descriptive summaries. Because each experimental condition in the fetal neural dataset included only one biological sample, pseudobulk differential expression testing was not performed. Cell numbers were not used as biological replicates.

### Exploratory differential expression analysis

Exploratory differential expression was assessed within each annotated lineage using cell-level Wilcoxon rank-sum tests implemented in Seurat FindMarkers on SCT-normalized values [23]. Because the fetal neural dataset included one biological sample for each experimental condition, these tests were used only as exploratory tools to rank genes and summarize descriptive transcriptional patterns. The resulting *P* values and FDR-adjusted values should not be interpreted as formal condition-level statistical inference, since cells from the same sample are not independent biological replicates and cannot estimate biological variance. Genes were therefore described as meeting exploratory differential expression criteria if they met |log_2_ fold change| ≥ 0.25, FDR < 0.05, and were detected in at least 10% of cells in one of the two groups. Comparisons included ZIKV-BR vs. mock, ZIKV-FSS vs. mock, and IFNβ vs. mock. Differences in the number of genes meeting these criteria were interpreted as descriptive condition-associated patterns and not as definitive evidence of strain-dependent effects.

### Interferon-stimulated gene (ISG) signature analysis

A curated panel of classical ISGs was prepared, including 11 canonical antiviral genes: IFITM1, MX1, OAS1, OAS2, OAS3, IFIT1, IFI6, ISG15, STAT1, IRF7, and RSAD2 [28–30]. Log_2_ fold-change values for these genes were taken for each lineage and contrast, row-scaled, and used to create supplementary heatmaps summarizing interferon-associated transcriptional patterns. These ISG summaries were interpreted as measures of transcriptional IFN/ISG responsiveness and were not used to infer IFNβ production, viral RNA burden, infection frequency, susceptibility, or cell-intrinsic antiviral initiation. Missing log_2_ fold-change values were kept as missing values and shown with a distinct visual indicator instead of being imputed as zero, so that missing entries were not read as absence of transcriptional change. Extreme values were trimmed only to improve visualization symmetry. Hierarchical clustering was used only to show descriptive similarities among genes; column order was fixed to keep interpretability across viral exposure and IFNβ conditions.

### ZIKV–IFNβ transcriptional similarity analysis

To assess transcriptional similarity between ZIKV-associated and IFNβ-associated responses, lineage-specific log_2_ fold-change vectors were compared between each ZIKV exposure condition and IFNβ stimulation. The main descriptive correlation analysis was performed using genes found in the exploratory differential expression tables for the compared contrasts. Because correlations based on filtered gene sets may be increased by shared ISG induction, sensitivity analyses were performed using both Pearson and Spearman correlations across several gene sets: all genes in the main exploratory differential expression tables, canonical ISGs only, non-ISG genes, and genes excluding the curated canonical ISG panel. An additional sensitivity analysis was performed using an expanded all-expressed/tested gene universe identified with log_2_ fold-change threshold = 0 and minimum detection threshold = 0.01 in Seurat FindMarkers. Canonical ISGs were annotated separately to distinguish ISG-driven similarity from non-ISG transcriptional similarity. All correlation analyses were interpreted descriptively and carefully rather than as evidence of functional equivalence between ZIKV-associated and IFNβ-stimulated programs.

### Independent bulk RNA-seq contextual analysis

An independent bulk RNA-seq dataset was used to give contextual support for interferon-associated transcriptional trends seen in the single-cell analyses, rather than as a lineage-resolved validation dataset. Processed RNA-seq count tables were obtained from the RNA-seq SubSeries GSE97919, which is part of the GSE104279 SuperSeries linked to Watanabe et al. [31]. This dataset includes bulk RNA-seq profiles from human cerebral organoid samples exposed to mock or ZIKV conditions and was used only as a bulk transcriptomic context for interferon-associated gene expression because it differs from the fetal neural single-cell dataset in experimental system, cellular composition, developmental model, and measurement modality. Differential expression analysis was performed using DESeq2. A generalized linear model was fitted, including organoid line and post-infection timepoint as covariates when available (design: ~ line + timepoint + condition). Genes with very low counts were filtered to improve model stability. Variance-stabilizing transformation (VST) was applied for visualization, principal component analysis (PCA), and sample-wise correlation analysis. To assess contextual concordance with the single-cell results, expression patterns of a curated panel of canonical interferon-stimulated genes (IFITM1, MX1, OAS1/2/3, IFIT1, IFI6, ISG15, STAT1, IRF7, RSAD2) were extracted and visualized as *z*-scored heatmaps.

### Contextual comparison using the Vero/moDC dataset

For the GSE230571 dataset, ISG module scores were calculated using AddModuleScore [23] with the same canonical ISG gene panel. Module scores were compared across Vero cells and primary human monocyte-derived dendritic cells and visualized on UMAP embeddings. This analysis was used only as a contextual comparison of interferon-associated transcriptional activation across different cellular systems. It was not interpreted as independent validation of fetal neural lineage-specific responses, radial glia-like responses, or cycling NPC-specific transcriptional patterns.

### Software

All analyses were performed in R (version 4.4.1) using Seurat (version 5.3.1). Additional packages used for data handling, visualization, and figure generation included ggplot2, dplyr, tidyr, readr, tibble, purrr, patchwork, ComplexHeatmap, circlize, ggplotify, DESeq2, ragg, magick, and scales. A random seed was set before ISG module scoring in the Vero/moDC contextual analysis. Unless otherwise stated, default package parameters were used. Key analysis parameters, including quality-control thresholds, normalization method, number of principal components, clustering resolution, exploratory differential expression thresholds, ISG gene list, and correlation sensitivity gene universes, are reported in the Methods and supplementary tables. Analysis scripts and derived outputs are available in the public repository described in the Data availability.

## Results

### Global transcriptional structure and dataset integrity

A total of 30,045 cells from human fetal brain-derived neural tissue and 67,666 cells from the Vero/monocyte-derived dendritic cell (moDC) system were analyzed. After quality control filtering, 27,267 fetal neural cells and 63,889 Vero/moDC cells were kept for downstream analyses (Supplementary Fig. S1). The fetal dataset included four experimental conditions—mock (5222 cells), ZIKV-BR (7814 cells), ZIKV-FSS (7966 cells), and IFNβ (6265 cells)—while the comparative dataset included ZIKV-infected Vero epithelial cells (42,429 cells) and primary dendritic cells (21,460 cells).

**Fig. 1:**
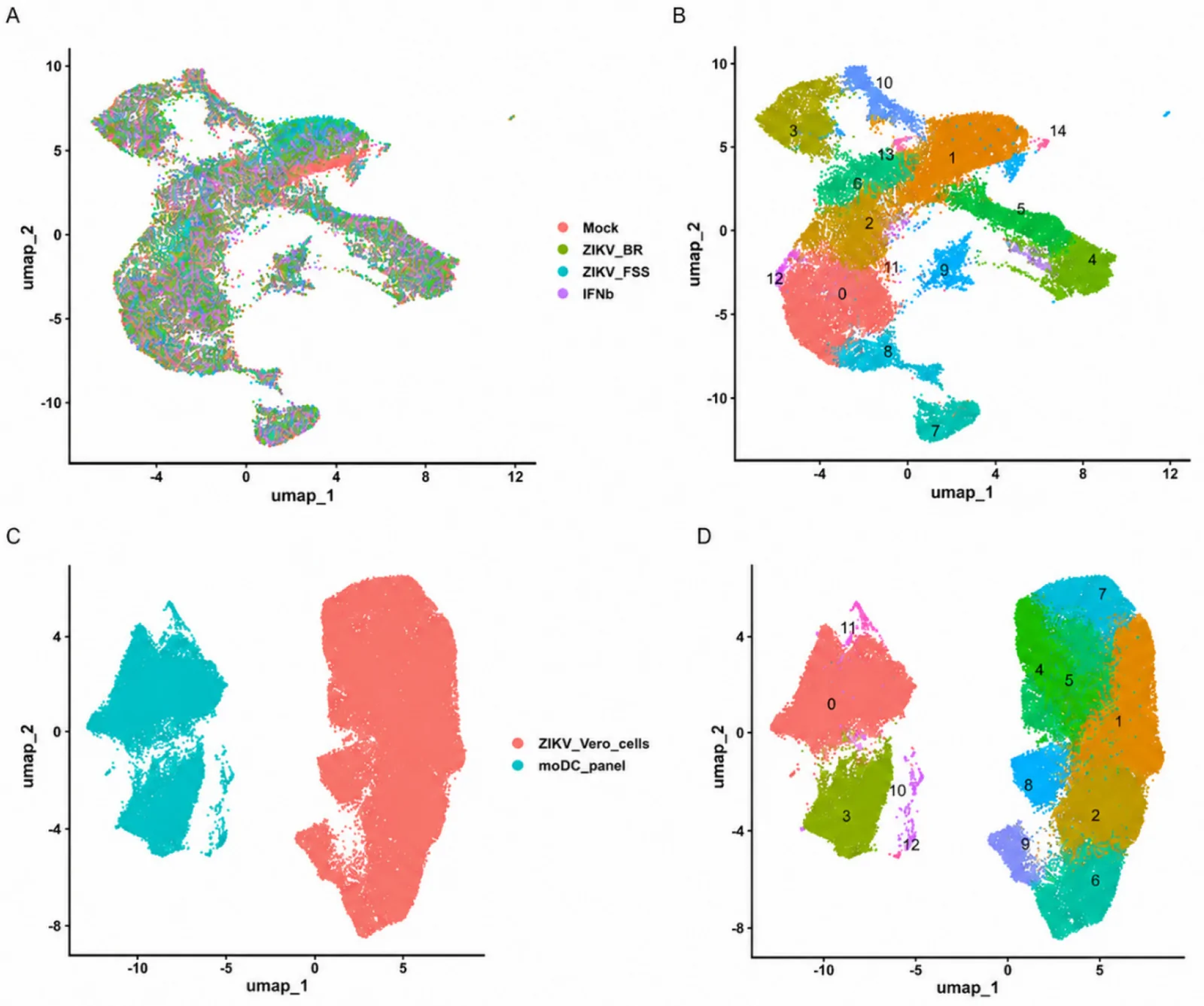
Global transcriptional structure of the fetal neural and Vero/moDC single-cell RNA-seq datasets. **A**, **B** UMAP embeddings of the GSE238140 fetal neural dataset colored by exposure condition and unsupervised Seurat cluster, respectively. Conditions included mock, ZIKV-BR, ZIKV-FSS, and IFNβ. **C**, **D** UMAP embeddings of the GSE230571 contextual Vero/moDC dataset colored by condition group and unsupervised Seurat cluster, respectively. UMAP coordinates are dimensionless embedding units. These visualizations were used descriptively to summarize dataset structure before downstream exploratory analyses

Dimensionality reduction using SCTransform normalization followed by principal component analysis (PCA) and UMAP embedding showed clear transcriptional organization across experimental conditions within the fetal brain samples (Fig. 1A, B). In the independent dataset, Vero cells and dendritic cells occupied separate regions of transcriptional space, consistent with their different cellular identities and innate immune capacities (Fig. 1C, D). Diagnostic elbow plots supported the use of 30 principal components for downstream analyses (Supplementary Fig. S1E, F), showing that the main sources of transcriptional variation were captured. Together, these results show clear dataset structure and provide a descriptive basis for later lineage-resolved analyses of antiviral transcriptional responses.

### Cellular architecture of fetal neural tissue under ZIKV exposure

**Fig. 2:**
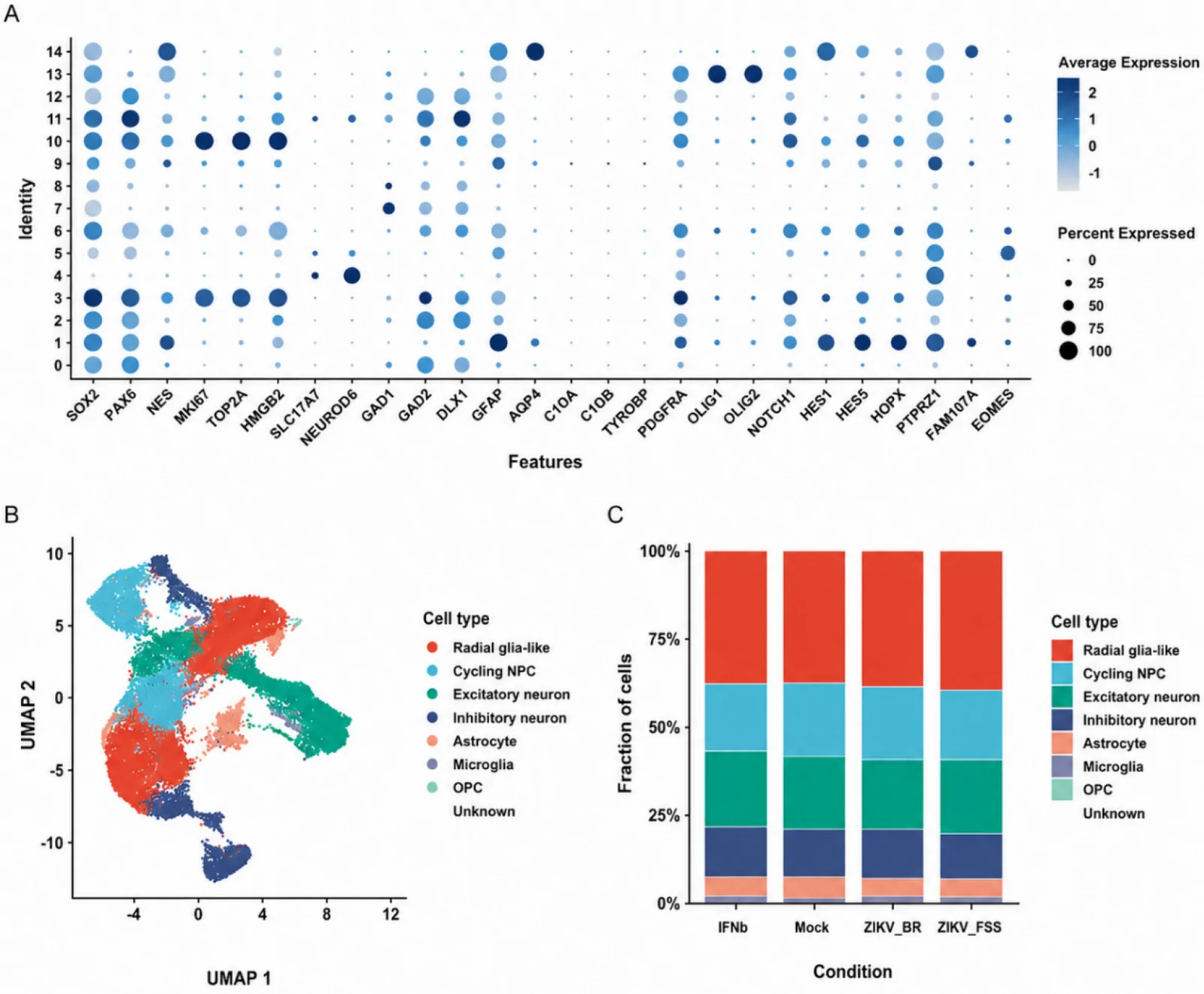
Cell-type architecture of the fetal neural scRNA-seq dataset under ZIKV exposure and IFNβ stimulation. **A** Dot plot showing canonical marker expression used to annotate major neural and glial populations in GSE238140. Dot size shows the percentage of cells expressing each marker, and color shows average scaled expression. **B** UMAP embedding colored by annotated cell type. The radial glia cluster was interpreted as a progenitor-enriched radial glia-like population because SOX2, PAX6, and NES are shared across progenitor states. **C** Descriptive cell-type composition across mock, ZIKV-BR, ZIKV-FSS, and IFNβ conditions. Cell-type proportions were summarized descriptively and were not interpreted as statistically powered condition-level differences

Unsupervised clustering resolved seven major neural and glial cell populations, including progenitor-enriched radial glia-like cells, cycling NPCs, excitatory neurons, inhibitory neurons, astrocytes, oligodendrocyte progenitor cells (OPCs), and microglia (Fig. 2A). The progenitor-enriched radial glia-like population formed the largest population (10,501 cells), followed by cycling NPCs (5459 cells), excitatory neurons (5670 cells), inhibitory neurons (3668 cells), astrocytes (1407 cells), microglia (506 cells), and a small OPC population (56 cells). The overall UMAP structure was consistent with expected developmental relationships, with progenitor populations located in central regions of the embedding and neuronal populations extending toward more differentiated transcriptional states (Fig. 2B).

Across experimental conditions, including ZIKV-BR, ZIKV-FSS, mock, and IFNβ stimulation, cell populations remained transcriptionally well defined, with differences in relative cell-type abundance observed between conditions (Fig. 2C). To summarize gene expression patterns at the lineage level while keeping sample identity, pseudobulk aggregation was performed, producing an expression matrix containing 23,183 genes across 28 lineage-specific condition combinations. This representation was used as a descriptive framework for later analyses of antiviral transcriptional responses.

### Lineage-specific transcriptional remodeling in response to viral and cytokine stimulation

**Fig. 3:**
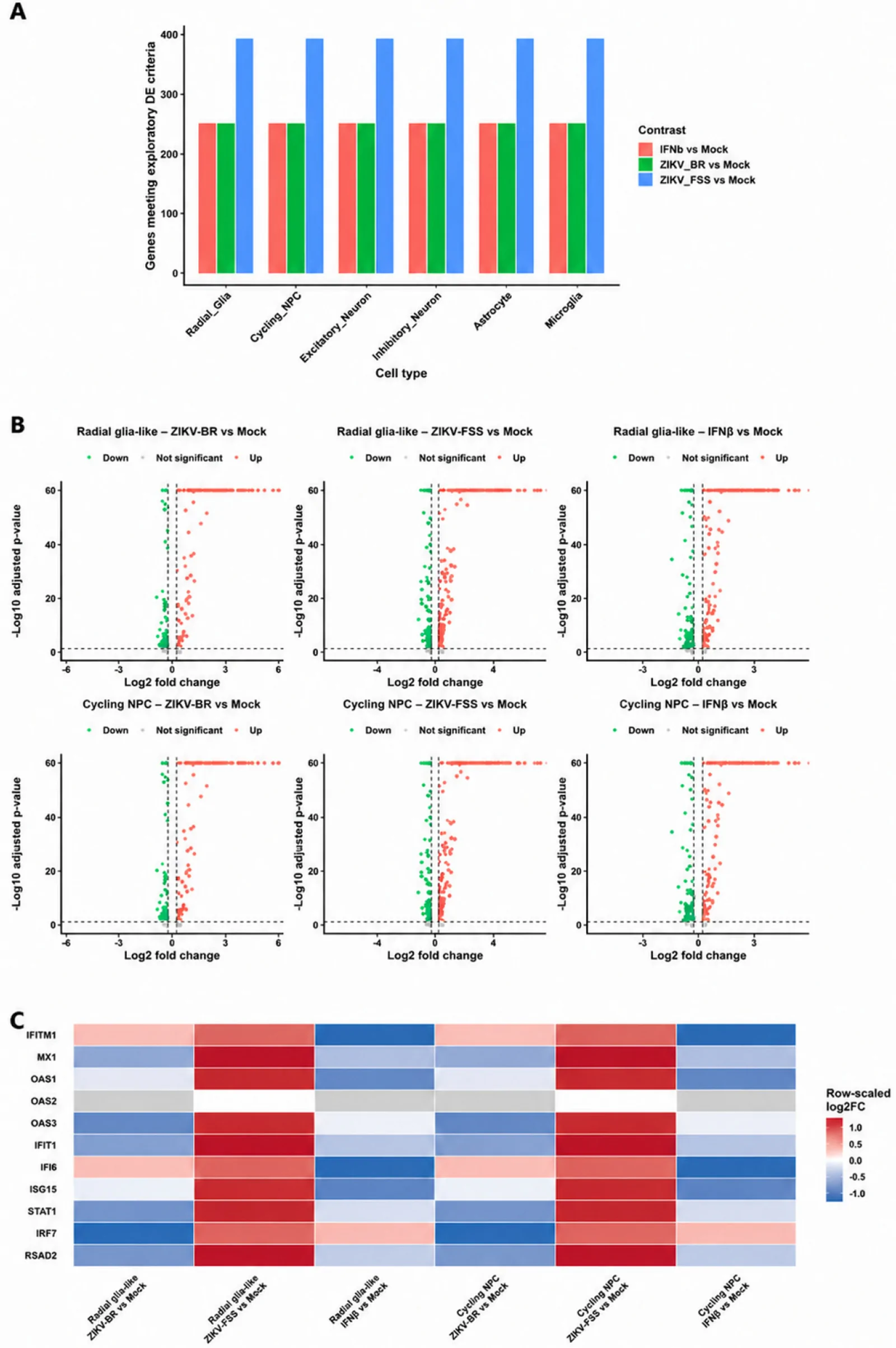
Lineage-specific and progenitor-focused exploratory transcriptional responses following ZIKV exposure or IFNβ stimulation. **A** Number of genes meeting exploratory differential expression criteria in each annotated fetal neural lineage after ZIKV-BR exposure, ZIKV-FSS exposure, or IFNβ stimulation compared with mock controls. **B** Volcano plots showing gene-level log_2_ fold-change patterns in progenitor-enriched radial glia-like and cycling NPC populations across the same contrasts. **C** Heatmap of row-scaled log_2_ fold-change values for a curated canonical ISG panel across progenitor-focused contrasts. All differential expression summaries were generated using cell-level Wilcoxon tests and are interpreted descriptively because each fetal neural condition was represented by one biological sample; cells were not treated as biological replicates

Exploratory differential expression analysis identified genes that met exploratory differential expression criteria across the examined neural lineages after ZIKV exposure or IFNβ stimulation (Fig. 3A). Progenitor-enriched radial glia-like, cycling NPC, astrocyte, and microglial populations showed a larger number of genes meeting these exploratory criteria compared with neuronal populations, while excitatory and inhibitory neurons showed fewer transcriptional changes. Across lineages, the number of genes meeting exploratory criteria differed among ZIKV-BR, ZIKV-FSS, and IFNβ conditions. Because these counts were derived from cell-level tests in a dataset with one sample per condition, they were used only to summarize observed transcriptional patterns and were not interpreted as evidence that one viral exposure condition produced a statistically broader response than another. Across conditions, interferon-stimulated genes (ISGs) were consistently induced, together with lineage-dependent variation in the expression of genes linked to stress and regulatory processes (Supplementary Fig. S2). Visualization of canonical ISG expression showed interferon-associated transcriptional responsiveness across neural populations, with the progenitor-enriched radial glia-like cluster showing strong relative ISG-associated expression patterns in this descriptive analysis (Supplementary Fig. S3). While both ZIKV strains and IFNβ stimulation produced overlapping antiviral transcriptional patterns, selected ISG-related and progenitor-focused summaries suggested greater descriptive similarity between ZIKV-FSS and IFNβ-associated profiles, whereas ZIKV-BR responses showed broader and more variable gene expression changes across lineages.

### Exploratory strain-associated transcriptional patterns in progenitor populations

Because progenitor-enriched radial glia-like and cycling NPC populations showed prominent transcriptional responses, these lineages were examined in more detail using gene-level volcano plots and a curated interferon-stimulated gene (ISG) heatmap (Fig. 3B, C). Exploratory comparisons suggested that progenitor-enriched radial glia-like and cycling NPC populations showed condition-associated differences in transcriptional response patterns between ZIKV-BR and ZIKV-FSS exposure. Because the fetal neural dataset included one biological sample per condition, these differences were interpreted as strain-associated exploratory patterns rather than definitive strain-dependent effects. These differences in gene counts were used as descriptive summaries of the observed transcriptional response patterns and not as statistically powered comparisons among cell types or viral exposure conditions. In selected progenitor-focused and ISG-related summaries, ZIKV-FSS exposure showed greater descriptive similarity to IFNβ-associated transcriptional patterns, whereas ZIKV-BR exposure showed broader and more variable transcriptional changes. Cycling NPCs also showed transcriptional IFN/ISG responsiveness across conditions, together with changes in selected cell-cycle-associated genes.

The canonical ISG heatmap supported interferon-associated transcriptional responsiveness across progenitor-focused contrasts, with ZIKV-FSS and IFNβ-associated profiles showing visually overlapping ISG-related patterns in this descriptive analysis (Fig. 3C). Because the analysis did not separate virus-positive cells from bystander cells or directly measure viral burden, these transcriptional patterns should not be interpreted as evidence of cell-intrinsic antiviral initiation, infection frequency, or functional susceptibility. Overall, these observations suggest that developmental identity and viral exposure condition were associated with differences in the structure and magnitude of transcriptional responses within progenitor populations, while not establishing definitive strain-dependent mechanisms.

### Comparative alignment between ZIKV-associated and IFNβ-associated transcriptional programs

**Fig. 4:**
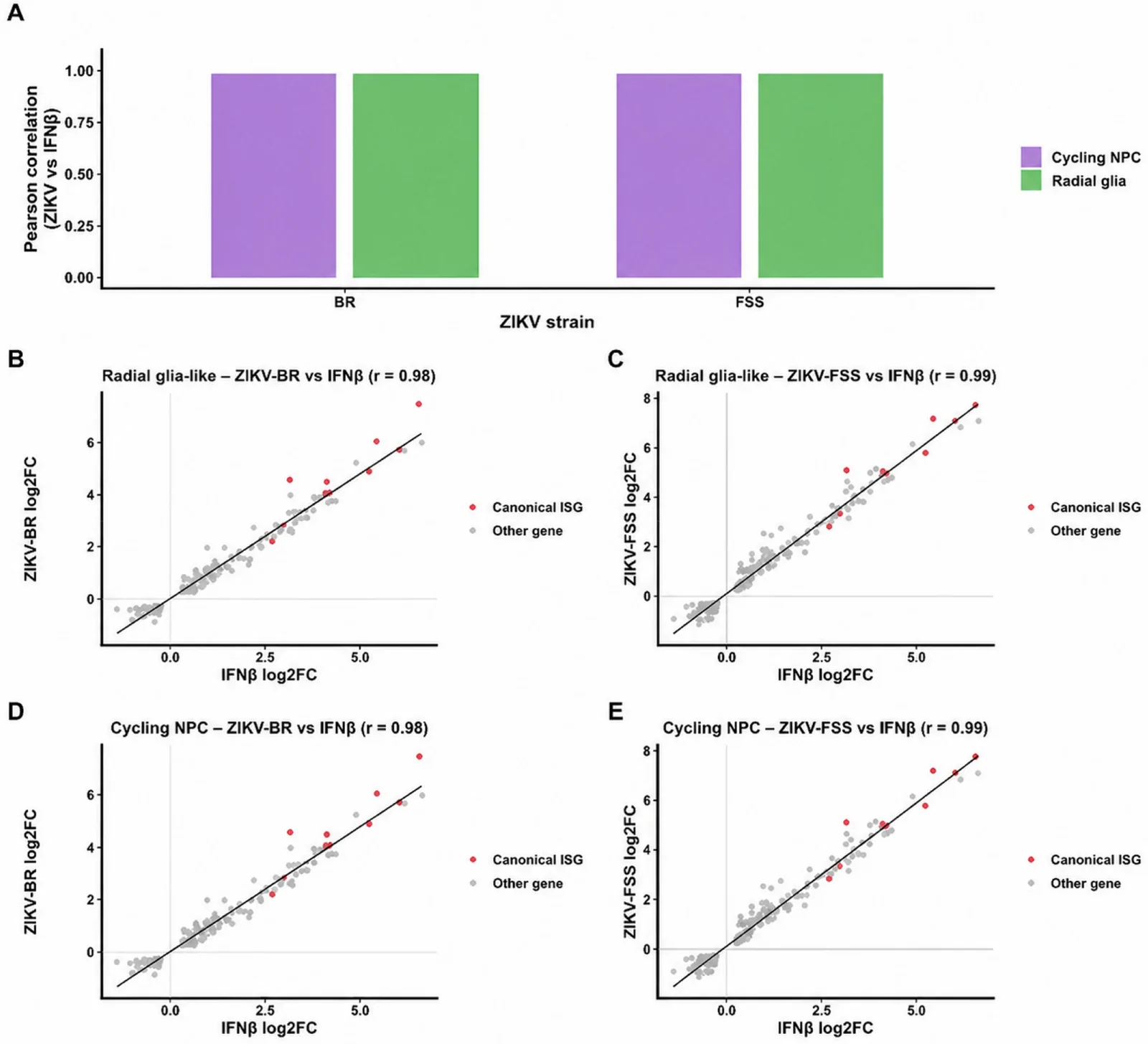
Descriptive transcriptional similarity between ZIKV-associated and IFNβ-associated responses. **A** Pearson correlation coefficients comparing IFNβ-associated and ZIKV-associated log_2_ fold-change patterns in progenitor-enriched radial glia-like and cycling NPC populations for ZIKV-BR and ZIKV-FSS exposure conditions. **B**–**E** Gene-level comparisons of log_2_ fold-change values, with IFNβ-associated responses on the x-axis and ZIKV-BR- or ZIKV-FSS-associated responses on the y-axis. Canonical interferon-stimulated genes (ISGs) are shown in red. Correlations are descriptive and were calculated from exploratory cell-level differential expression summaries; they should not be interpreted as formal condition-level inference or as evidence of functional equivalence between ZIKV-associated and IFNβ-stimulated programs. Sensitivity analyses using Pearson and Spearman correlations across primary DE-table genes, all-expressed/tested genes, canonical ISGs, non-ISG genes, and ISG-excluded gene sets are provided in Supplementary Table 1

We next assessed whether ZIKV-associated transcriptional changes were similar to IFNβ-associated responses within progenitor-enriched radial glia-like and cycling NPC populations. In the main descriptive analysis based on genes present in the exploratory differential expression tables, ZIKV–IFNβ log_2_ fold-change correlations were high, consistent with broad overlap in interferon-associated transcriptional programs (Fig. 4A–E). However, sensitivity analyses showed that correlation estimates strongly depended on the gene universe used (Supplementary Fig. S5 and Supplementary Table 1). Correlations calculated from the primary exploratory DE-table genes were consistently high across canonical ISG, non-ISG, and ISG-excluded gene classes, indicating that DEG-table filtering can overestimate apparent ZIKV–IFNβ transcriptional similarity. When correlations were recalculated across the expanded all-expressed/tested gene universe, Pearson correlations were lower and Spearman correlations were much more conservative, especially for non-ISG and ISG-excluded genes. Canonical ISGs showed strong concordance between ZIKV exposure and IFNβ stimulation, whereas non-ISG and ISG-excluded genes gave a broader and more conservative estimate of ZIKV–IFNβ alignment. Therefore, ZIKV–IFNβ correlations were interpreted as descriptive evidence of shared IFN/ISG responsiveness and broader transcriptional alignment, rather than functional equivalence between virus-associated and cytokine-stimulated programs. Full Pearson and Spearman correlation summaries across gene universes and gene classes are provided in Supplementary Table 1.

### Transcriptional response magnitude across developmental identities

**Fig. 5:**
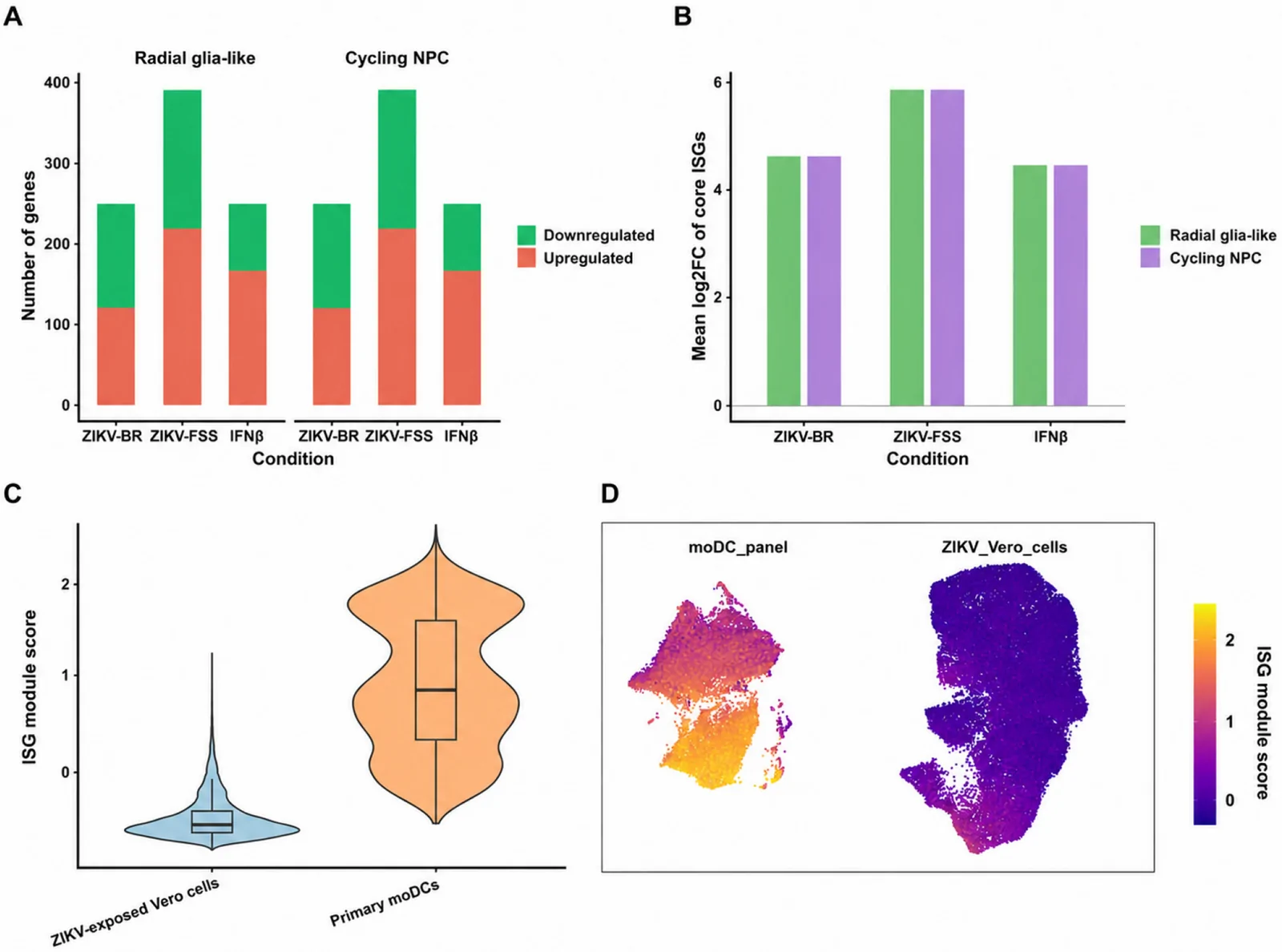
Contextual comparison of IFN/ISG-associated transcriptional patterns across fetal neural and Vero/moDC datasets. **A** Genes meeting exploratory differential expression criteria in progenitor-enriched radial glia-like and cycling NPC populations from GSE238140 after ZIKV-BR, ZIKV-FSS, or IFNβ exposure compared with mock controls. **B** Mean log_2_ fold-change of the curated core ISG panel across the same neural progenitor populations. **C** ISG module scores in the contextual GSE230571 Vero/moDC dataset, comparing ZIKV-exposed Vero cells and primary human moDCs. **D** UMAP projection of the Vero/moDC dataset colored by ISG module score. These analyses summarize transcriptional IFN/ISG responsiveness and contextual cross-system patterns, not IFNβ production, viral burden, infection frequency, susceptibility, or antiviral efficacy

Quantitative summaries of differential gene expression and interferon-stimulated gene (ISG) induction showed differences in the magnitude of transcriptional responses across neural lineages (Fig. 5A, B). Across perturbations, the progenitor-enriched radial glia-like cluster showed a higher number of genes meeting exploratory differential expression criteria compared with several other cell types, while cycling NPCs showed condition-associated ISG responsiveness and changes in selected cell-cycle-associated genes. These summaries describe transcriptional response magnitude and IFN/ISG responsiveness; they do not measure IFNβ production, viral RNA burden, infection frequency, susceptibility, or antiviral efficacy. Notably, ZIKV exposure was associated with detectable induction of antiviral-associated transcripts in progenitor populations, even without exogenous cytokine stimulation. These observations suggest that developmental identity is linked to differences in the scale of antiviral transcriptional responses observed across conditions, without implying functional susceptibility or antiviral efficacy.

### Independent bulk RNA-seq contextual analysis

To put the interferon-associated transcriptional patterns observed at single-cell resolution into a wider context, we analyzed the independent bulk RNA-seq SubSeries GSE97919, part of the GSE104279 SuperSeries associated with Watanabe et al. [31]. This dataset includes bulk RNA-seq profiles from human cerebral organoid samples exposed to Mock or ZIKV conditions and was used only as a contextual bulk transcriptomic comparison. Principal component analysis of variance-stabilized expression values showed partial separation between ZIKV-exposed and control samples, consistent with a detectable transcriptional response to ZIKV exposure at the bulk level (Fig. S4A). Across samples and timepoints, canonical interferon-stimulated genes showed coordinated upregulation after ZIKV exposure (Fig. S4B). Differential expression analysis identified induction of established antiviral genes in infected samples compared with controls (Fig. S4C), and sample-wise correlation analysis showed high within-condition concordance (Fig. S4D). Although bulk RNA-seq does not resolve lineage-specific responses, these data provide contextual tissue-level support for the broad occurrence of interferon-associated transcriptional activation in ZIKV-exposed cerebral organoid samples and were not interpreted as validation of fetal neural cell-type-specific patterns.

### Contextual comparison across immune and epithelial cell models

In an independent dataset including ZIKV-infected monocyte-derived dendritic cells and Vero epithelial cells, ISG module scoring showed strong interferon-associated transcriptional activation in dendritic cells, with only minimal induction in Vero cells (Fig. 5C, D). ISG enrichment was located in the dendritic cell compartment in UMAP projections, consistent with known differences in innate immune competence between these cell types. Together, these observations provide contextual support for broad IFN/ISG-associated transcriptional activation in ZIKV-exposed systems with intact innate immune signaling. However, because the fetal neural single-cell, cerebral organoid bulk RNA-seq, immune, and epithelial datasets differ in species, cell-type composition, experimental design, infection context, and measurement modality, these cross-dataset comparisons were not interpreted as validation of fetal neural lineage-specific responses.

## Discussion

Congenital Zika syndrome (CZS) shows the close interaction between viral infection and early human neurodevelopment, but the transcriptional features behind lineage-specific host responses are still not fully characterized [32–34]. Rather than presenting a new primary atlas or mechanistic analysis of ZIKV pathogenesis, this study provides a careful lineage-focused secondary reanalysis of public transcriptomic datasets. The main fetal neural dataset was previously generated and deeply analyzed by Stokes et al. [11]; therefore, the contribution of the present work is to provide a standardized descriptive summary of cell-type-level transcriptional response patterns, clearly frame differential expression results as exploratory because of limited biological replication, and assess the sensitivity of ZIKV–IFNβ transcriptional similarity to gene-set selection. The observation of ISG induction across different datasets provides contextual support for the broad presence of interferon-associated transcriptional programs during ZIKV exposure [11], but does not validate fetal neural lineage-specific responses.

Across conditions, progenitor-enriched radial glia-like and cycling NPC populations showed clear transcriptional IFN/ISG responsiveness and differences in the number of genes meeting exploratory differential expression criteria compared with several other neural lineages. These observations describe transcriptional response magnitude rather than IFNβ production, viral RNA burden, infection frequency, or functional susceptibility. This distinction is important because the present analysis did not separate virus-positive cells from bystander cells and therefore cannot determine whether ISG induction in progenitor populations reflects cell-intrinsic antiviral initiation or paracrine IFN/IFNAR-mediated signaling. This interpretation is consistent with the mechanistic framework of Stokes et al. [11] in which astrocytes were highlighted as IFNβ-producing sentinel cells, while ISG expression in progenitor populations may reflect IFN responsiveness or bystander signaling rather than autonomous antiviral initiation. The observation that progenitor-enriched radial glia-like and cycling NPC populations also showed IFN/ISG-associated transcriptional responses after exogenous IFNβ stimulation in the present analysis further supports their interpretation as IFN-responsive populations rather than as demonstrated initiators of cell-intrinsic antiviral signaling. These differences in response magnitude are consistent with previous observations that early progenitor populations undergo dynamic transcriptional changes during human cortical development [27, 34]. The increased ISG-associated expression patterns and the larger number of genes meeting exploratory differential expression criteria in progenitor-enriched populations observed in the present analysis suggest descriptive variation in transcriptional response magnitude across developmental states, without supporting formal condition-level inference or causal conclusions.

Viral exposure condition was also linked to exploratory differences in transcriptional response structure. Although both ZIKV-BR and ZIKV-FSS exposure were associated with overlapping interferon-related transcriptional patterns, the two viral conditions showed differences in the number and composition of genes meeting exploratory differential expression criteria. Because the fetal neural dataset included one biological sample per condition [11], these observations should be interpreted as strain-associated descriptive patterns rather than definitive evidence of strain-dependent mechanisms. The present analysis does not determine whether these differences reflect viral genotype, infection efficiency, viral RNA burden, donor or tissue effects, cell-type composition, sample handling, or other experimental factors.

A central observation of this study is the partial overlap, together with gene-universe-dependent divergence, between ZIKV-associated and IFNβ-associated transcriptional responses. Canonical interferon-stimulated genes showed strong concordance across viral and cytokine conditions, but sensitivity analyses showed that correlation estimates were strongly affected by the gene set used for comparison. Correlations calculated from primary exploratory DE-table genes remained high even after excluding the curated canonical ISG panel, while correlations across the expanded all-expressed/tested gene universe were lower, especially when assessed using Spearman correlation. These findings suggest that ZIKV-associated and IFNβ-associated responses share broad transcriptional alignment but should not be interpreted as functionally equivalent or as evidence of one IFNβ-driven program. The present analyses are descriptive and do not establish regulatory interactions or signaling hierarchies underlying these patterns [29].

The Vero/moDC analysis was included only as a contextual comparison across different cellular systems. The observation that monocyte-derived dendritic cells showed stronger ISG module scores than Vero cells is consistent with differences in innate immune competence between these systems [16], but this comparison does not validate fetal neural lineage-specific findings. Instead, it supports the broader interpretation that ZIKV exposure can be accompanied by interferon-associated transcriptional programs in cell types with intact innate immune signaling, while the magnitude and structure of these responses remain dependent on cellular context and experimental design.

Several limitations should be considered when interpreting these results. First, the ex vivo exposure conditions analyzed here may not fully represent the complexity of in vivo ZIKV infection. Second, the lack of biological replication for each experimental condition limits formal statistical inference, and differential expression analyses should therefore be interpreted as exploratory. In addition, the progenitor-enriched radial glia-like cluster was not separated into apical radial glia, outer/basal radial glia, or intermediate progenitor subtypes; therefore, findings for this cluster should be interpreted as mixed progenitor-enriched transcriptional patterns rather than subtype-specific radial glia responses. Finally, differences in species, cell-type composition, experimental design, infection context, and measurement modality limit direct comparisons between fetal neural scRNA-seq, cerebral organoid bulk RNA-seq, Vero cells, and moDC datasets; therefore, cross-dataset analyses were used only for contextual comparison and not for validation of fetal neural lineage-specific responses.

## Conclusion

In this study, we present an exploratory lineage-resolved secondary analysis of publicly available single-cell transcriptomic data from developing human neural tissue after exposure to ZIKV and IFNβ. Across annotated neural lineages, progenitor-enriched radial glia-like and cycling NPC populations showed strong IFN/ISG-associated transcriptional responsiveness and a larger number of genes meeting exploratory differential expression criteria compared with several other cell types. Exploratory comparisons suggested strain-associated differences in transcriptional response patterns between ZIKV-BR and ZIKV-FSS, with partial overlap observed between ZIKV-associated and IFNβ-associated gene expression profiles. While canonical interferon-stimulated genes were consistently induced across conditions, additional gene expression changes associated with viral exposure were not fully reproduced by IFNβ stimulation alone, particularly when broader non-ISG or all-expressed gene universes were considered. Together, these findings provide a descriptive, lineage-resolved reference of antiviral transcriptional states in developing human neural tissue and show how developmental identity and viral exposure condition are associated with variation in host transcriptional responses to ZIKV exposure. Because the primary fetal neural dataset contained one biological sample per condition, these results should be interpreted as exploratory strain-associated patterns rather than definitive evidence of strain-dependent mechanisms.

## Supplementary Information


Supplementary Material 1.

## Data Availability

All datasets analyzed in this study are publicly available from the Gene Expression Omnibus under accession numbers GSE238140, GSE230571, and GSE97919. The R analysis scripts, curated sample metadata tables, gene lists, and documentation required to reproduce the analyses have been deposited in a public GitHub repository at https://github.com/abedizahra/Zika-scrnaseq-antiviral-response-code. Raw sequencing data were not deposited in the repository because they are publicly available through GEO. Derived results can be regenerated by running the scripts according to the workflow described in the repository README.
