# Supplementary material for: Exploratory strain-associated patterns of antiviral transcriptional responses to Zika virus exposure in developing human neural tissue
Source: Genomics Inform. 2026 Jul 9;24:14. doi: 10.1186/s44342-026-00076-5 (PMC13352994; doi:10.1186/s44342-026-00076-5)
Supplement: Supplementary file 1 — Supplementary Material 1. [file 44342_2026_76_MOESM1_ESM.docx]

**Exploratory strain-associated patterns of antiviral transcriptional responses to Zika virus exposure in developing human neural tissue**

Zahra Abedi^1*^, [Mohammad Ali Sheikh Beig Goharrizi](https://pubmed.ncbi.nlm.nih.gov/?sort=date&term=Sheikh+Beig+Goharrizi+MA&cauthor_id=35393513)^2*^, Amirreza Abbasi^3^, Negar Sadat Soleimani Zakeri^4,^ Helia Jangi^5^, Alireza Susanabadi Farahani^6^

*^1^ Dina pharmed Exir Salamat, Pharmaceutical Co., 574768, Tehran, Iran.*

*^2^ Department of Medical Biotechnology, School of Biotechnology, College of Science, University of Tehran, Tehran, Iran.*

*^3^ Department of Biology, Azad University of Tabriz, Tabriz, IRAN.*

*^4^ Department of Software Engineering, Engineering and Architecture Faculty, Istanbul Nişantaşi University, Istanbul, Turkey.*

*^5^ Department of Biology, Ferdowsi University of Mashhad, Mashhad, IRAN.*

*^6^ Department of Anesthesia and pain, Arak University of medical sciences, Arak, Iran*.

**ORCID IDs:**

Zahra Abedi: 0000-0002-6750-0208

Mohammad Ali Sheikh Beig Goharrizi: 0000-0001-7280-6175

Amirreza Abbasi: 0000-0001-5437-679X

Negar SadatSoleimaniZakeri: 0000-0002-3343-9904

Helia Jangi: 0000-0007-8782-6989

Alireza Susanabadi Farahani: 0009-0001-6813-3059

***Correspondence Authors**

| **Zahra Abedi.**  Dina pharmed Exir Salamat, Pharmaceutical Co., 574768, Tehran, Iran.  E-mail: [abedizahra59@yahoo.com](mailto:abedizahra59@yahoo.com) | **Mohammad Ali Sheikh Beig Goharrizi.**  Department of Medical Biotechnology, School of Biotechnology, College of Science, University of Tehran, Tehran, Iran.  E-mail: msheikhbeig@ut.ac.ir |
| --- | --- |


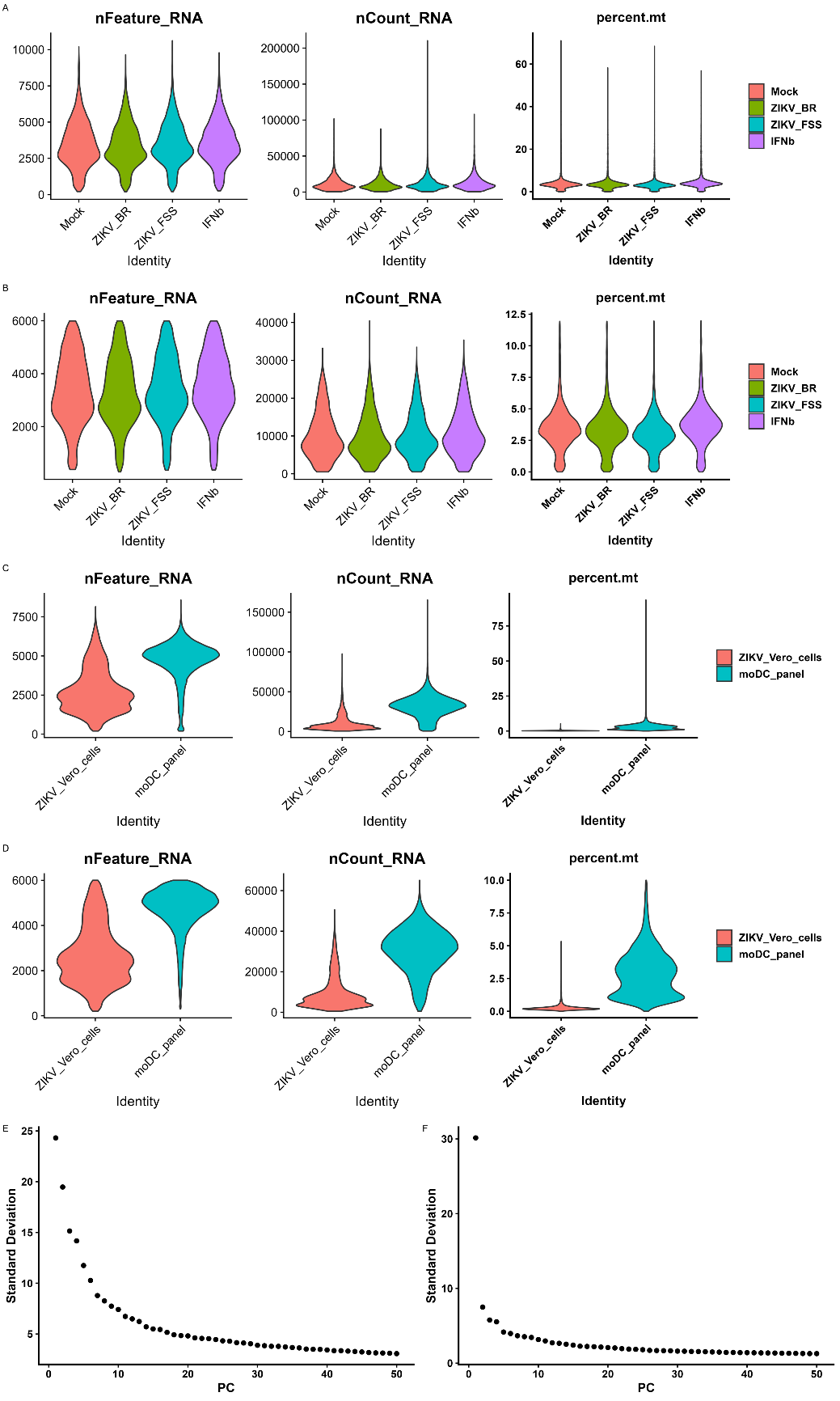


**Figure S1.** **Quality control and dimensionality assessment of scRNA-seq datasets.** (A–B) Violin plots showing distributions of the number of detected genes per cell (nFeature_RNA), total UMI counts per cell (nCount_RNA), and the percentage of mitochondrial gene expression (percent.mt) across experimental conditions (Mock, ZIKV-BR, ZIKV-FSS, and IFNβ) in the human fetal brain organoid dataset before and after quality control filtering.
(C–D) Corresponding quality control metrics for the independent ZIKV-infected Vero cell and monocyte-derived dendritic cell (moDC) dataset, illustrating differences in sequencing depth and mitochondrial content between epithelial and immune cell populations. (E–F) Elbow plots displaying the standard deviation of principal components (PCs) for the fetal brain organoid dataset (E) and the Vero/moDC dataset (F), supporting the selection of the top 30 principal components for downstream dimensionality reduction and clustering analyses.


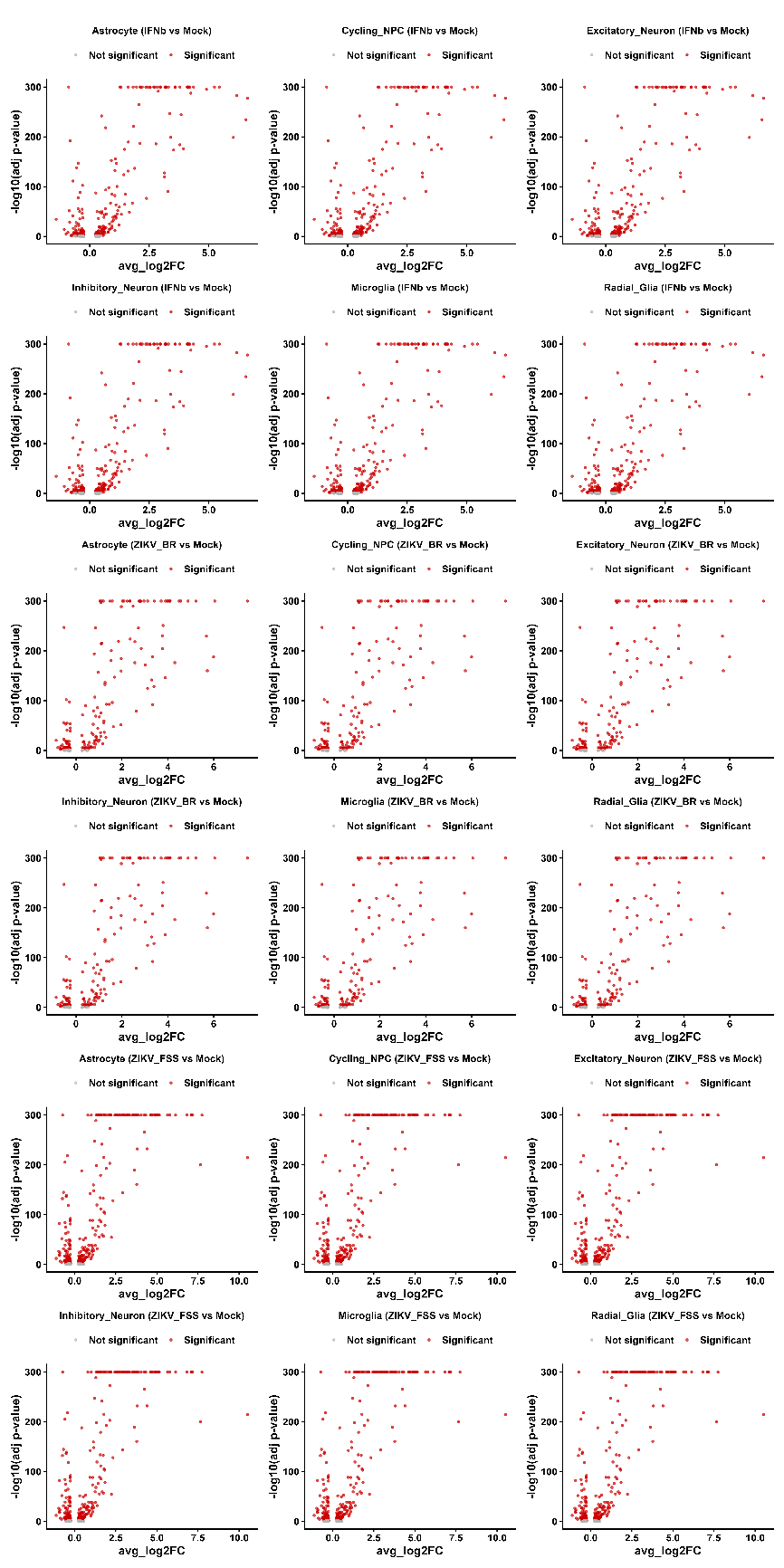


**Figure S2. Lineage-specific differential gene expression following IFNβ stimulation and ZIKV infection.**
Volcano plots showing differential gene expression across neural lineages (astrocytes, cycling neural progenitors, excitatory neurons, inhibitory neurons, microglia, and radial glia) following exposure to IFNβ, ZIKV-BR, or ZIKV-FSS, each compared to mock-treated controls. Each dot represents a gene, plotted by average log₂ fold change (x-axis) and −log₁₀(adjusted p-value) (y-axis). Significantly differentially expressed genes (Wilcoxon rank-sum test; FDR < 0.05 and |log₂ fold change| ≥ 0.25) are highlighted in red, while non-significant genes are shown in gray. Across conditions, progenitor populations—particularly radial glia and cycling neural progenitors—exhibit a higher density of significantly altered genes, reflecting enhanced transcriptional responsiveness relative to more differentiated neuronal lineages.


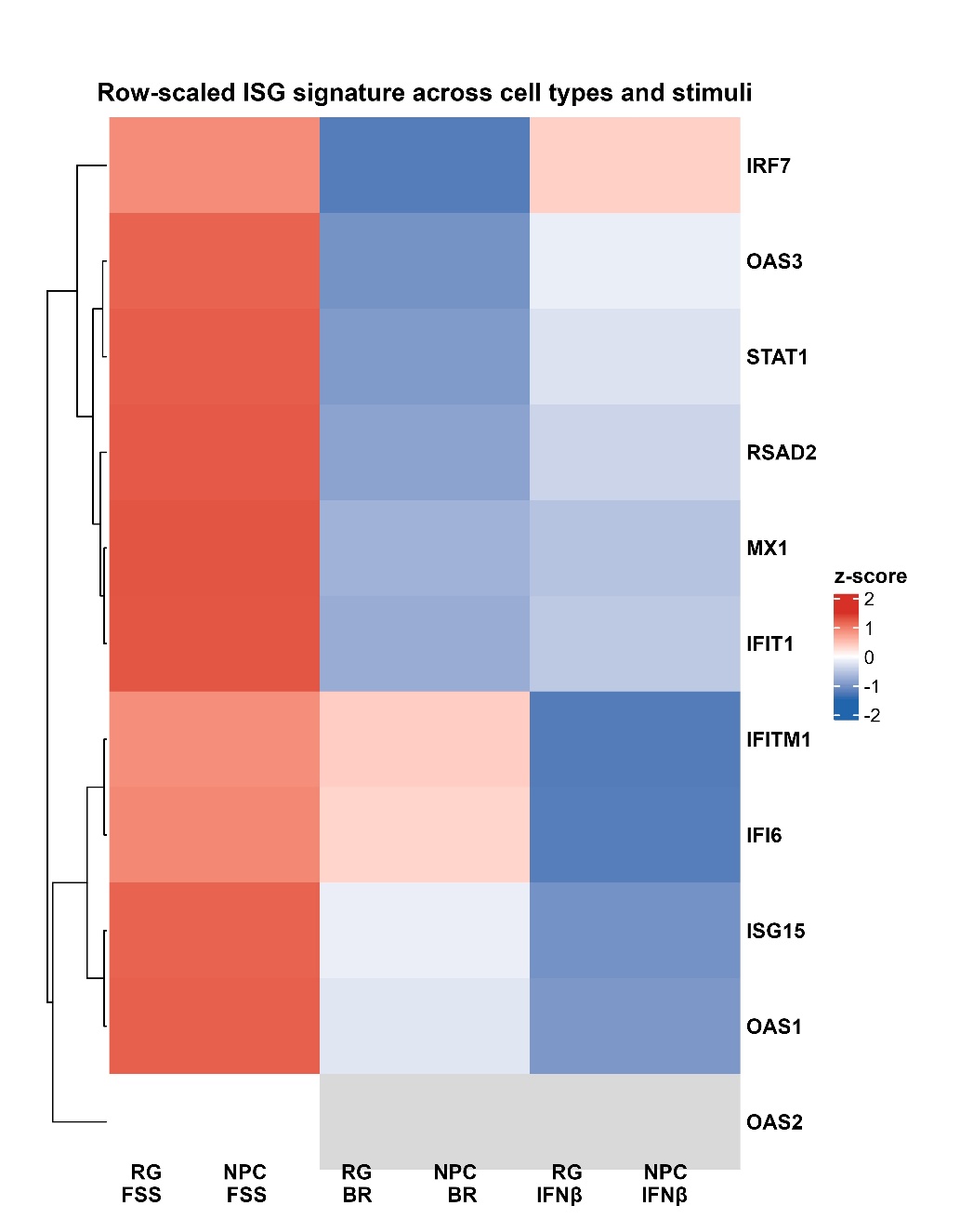


**Figure S3.** **Canonical ISG-associated transcriptional patterns across neural lineages and exposure conditions.** Heatmap showing row-scaled log₂ fold-change values for the curated canonical ISG panel across annotated fetal neural lineages following ZIKV-BR exposure, ZIKV-FSS exposure, or IFNβ stimulation relative to mock controls. Values were derived from exploratory cell-level differential expression summaries and are shown only as descriptive measures of transcriptional IFN/ISG responsiveness. Missing ISG–lineage–condition combinations were retained as missing values and displayed with a distinct visual indicator rather than being imputed as zero; therefore, blank or gray entries should not be interpreted as absence of fold-change. Because the fetal neural dataset contained one biological sample per condition, these patterns should not be interpreted as formal condition-level statistical inference. Four ISG–lineage–condition combinations were missing from the exploratory differential-expression tables; these entries were retained as missing values, listed in ISG_heatmap_missing_values.csv, and displayed in gray rather than being imputed as zero.


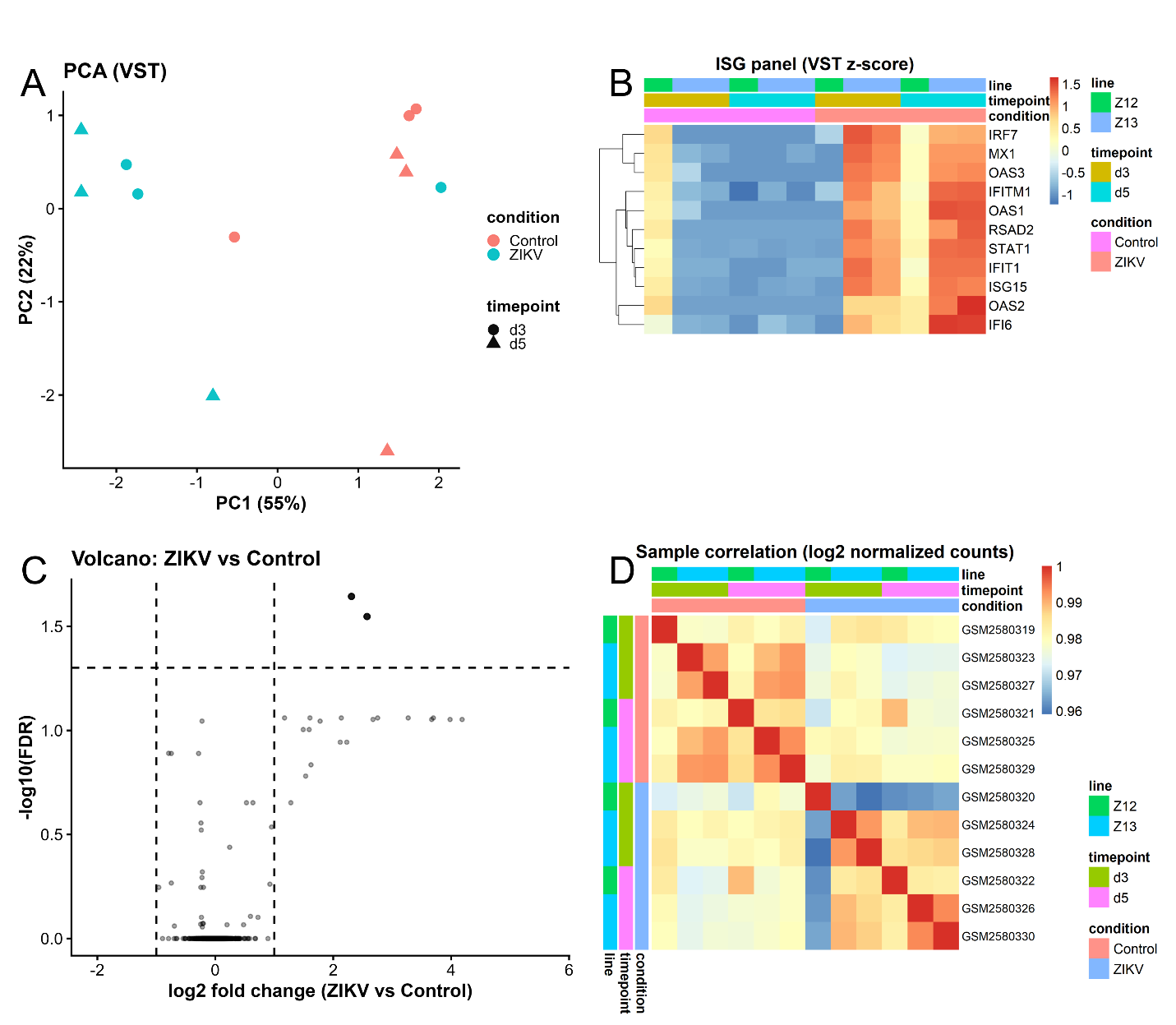


**Figure S4. Independent bulk RNA-seq contextual support for interferon-associated transcriptional responses.**
(A) PCA of variance-stabilized bulk RNA-seq profiles from human cerebral organoid samples exposed to mock or ZIKV conditions in the GSE97919 RNA-seq SubSeries, part of the GSE104279 SuperSeries associated with Watanabe et al. (B) Z-scored heatmap of canonical interferon-stimulated genes (ISGs) across samples, illustrating coordinated antiviral gene induction upon ZIKV exposure. (C) Volcano plot showing differential gene expression between ZIKV-exposed and control samples. (D) Sample-wise correlation heatmap based on log₂-normalized counts. This bulk RNA-seq analysis was used only as a contextual tissue-level comparison of interferon-associated transcriptional signatures and does not provide lineage-specific resolution or validation of fetal neural cell-type-specific responses.


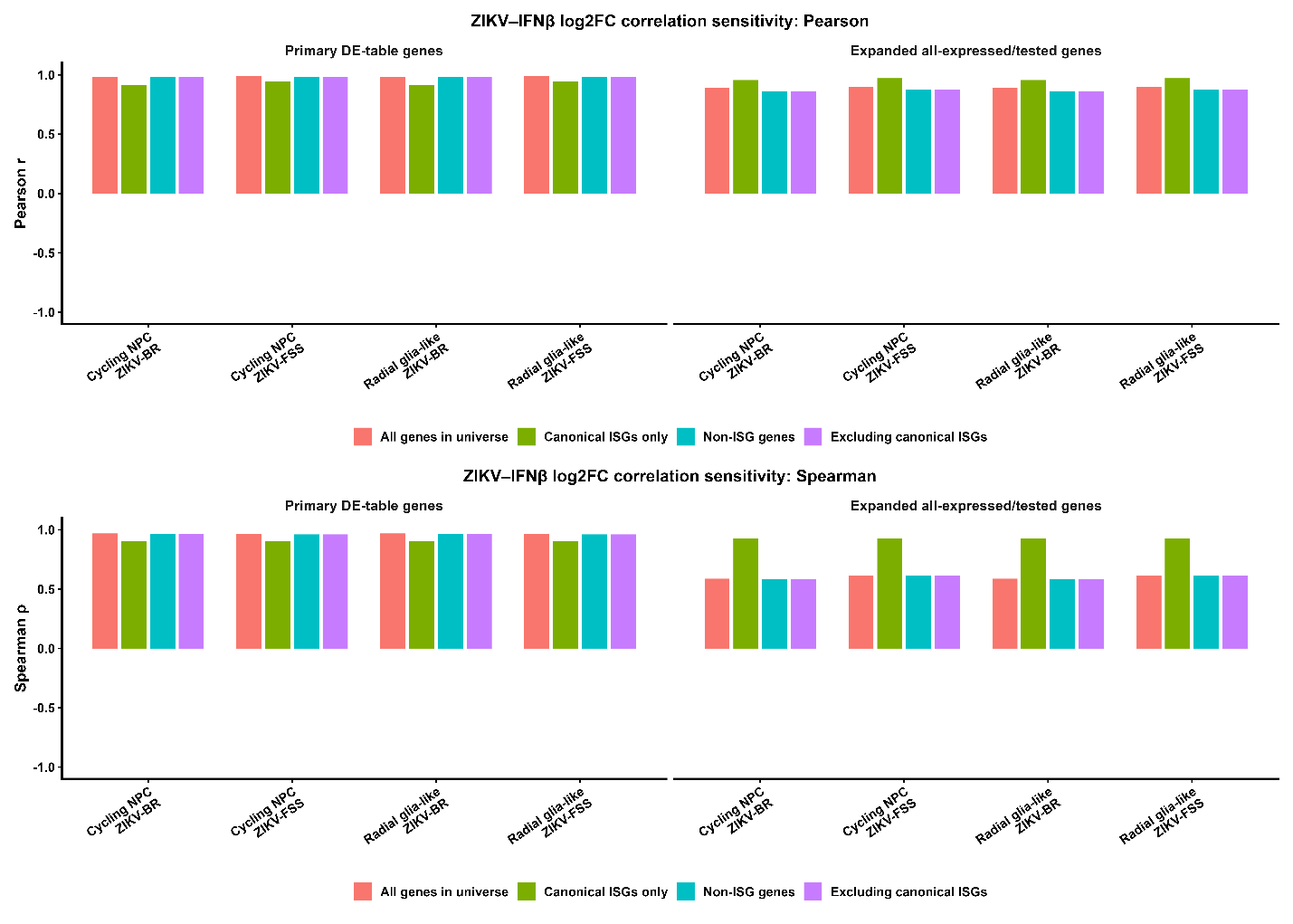


**Figure S5. Sensitivity analysis of ZIKV–IFNβ transcriptional similarity across gene universes and gene classes.**
Pearson and Spearman correlations were calculated between ZIKV-associated and IFNβ-associated log₂ fold-change vectors in progenitor-enriched radial glia-like and cycling NPC populations. Correlations were compared across the primary exploratory differential-expression table genes and an expanded all-expressed/tested gene universe, and stratified by all genes, canonical ISGs, non-ISG genes, and genes excluding the curated canonical ISG panel. The analysis shows that ZIKV–IFNβ similarity estimates depend strongly on gene-universe selection and should be interpreted descriptively rather than as evidence of functional equivalence between virus-associated and cytokine-stimulated programs.

**Supplementary Table 1. Sensitivity analysis of ZIKV–IFNβ transcriptional similarity across gene universes and gene classes.** Pearson and Spearman correlations were calculated between ZIKV-associated and IFNβ-associated log₂ fold-change vectors in progenitor-enriched radial glia-like and cycling NPC populations. Correlations were calculated using primary exploratory DE-table genes and an expanded all-expressed/tested gene universe. Within each universe, correlations were stratified across all genes, canonical ISGs only, non-ISG genes, and genes excluding the curated canonical ISG panel. These analyses show that correlation estimates are sensitive to gene-universe selection and should be interpreted descriptively rather than as evidence of functional equivalence between ZIKV-associated and IFNβ-associated programs.

| **Comparison** | **Cell_type** | **Zikv_condition** | **Gene_universe** | **Gene_class** | **n_genes** | **Pearson_r** | **Spearman_r** |
| --- | --- | --- | --- | --- | --- | --- | --- |
| NPC_BR | Cycling NPC | BR | all_expressed_genes_minpct_0.01 | all_genes_in_universe | 14166 | 0.889696968 | 0.587198564 |
| NPC_BR | Cycling NPC | BR | all_expressed_genes_minpct_0.01 | canonical_ISGs_only | 11 | 0.955518776 | 0.927272727 |
| NPC_BR | Cycling NPC | BR | all_expressed_genes_minpct_0.01 | excluding_canonical_ISGs | 14155 | 0.863891618 | 0.586235442 |
| NPC_BR | Cycling NPC | BR | all_expressed_genes_minpct_0.01 | non_ISG_genes | 14155 | 0.863891618 | 0.586235442 |
| NPC_BR | Cycling NPC | BR | primary_exploratory_DE_tables | all_genes_in_universe | 191 | 0.983728031 | 0.969337283 |
| NPC_BR | Cycling NPC | BR | primary_exploratory_DE_tables | canonical_ISGs_only | 10 | 0.915819639 | 0.903030303 |
| NPC_BR | Cycling NPC | BR | primary_exploratory_DE_tables | excluding_canonical_ISGs | 181 | 0.984380504 | 0.964725882 |
| NPC_BR | Cycling NPC | BR | primary_exploratory_DE_tables | non_ISG_genes | 181 | 0.984380504 | 0.964725882 |
| NPC_FSS | Cycling NPC | FSS | all_expressed_genes_minpct_0.01 | all_genes_in_universe | 14176 | 0.899836318 | 0.613552483 |
| NPC_FSS | Cycling NPC | FSS | all_expressed_genes_minpct_0.01 | canonical_ISGs_only | 11 | 0.972122258 | 0.927272727 |
| NPC_FSS | Cycling NPC | FSS | all_expressed_genes_minpct_0.01 | excluding_canonical_ISGs | 14165 | 0.877363387 | 0.612651487 |
| NPC_FSS | Cycling NPC | FSS | all_expressed_genes_minpct_0.01 | non_ISG_genes | 14165 | 0.877363387 | 0.612651487 |
| NPC_FSS | Cycling NPC | FSS | primary_exploratory_DE_tables | all_genes_in_universe | 258 | 0.986839438 | 0.965453257 |
| NPC_FSS | Cycling NPC | FSS | primary_exploratory_DE_tables | canonical_ISGs_only | 10 | 0.943701024 | 0.903030303 |
| NPC_FSS | Cycling NPC | FSS | primary_exploratory_DE_tables | excluding_canonical_ISGs | 248 | 0.984444168 | 0.961385047 |
| NPC_FSS | Cycling NPC | FSS | primary_exploratory_DE_tables | non_ISG_genes | 248 | 0.984444168 | 0.961385047 |
| RG_BR | Radial glia-like | BR | all_expressed_genes_minpct_0.01 | all_genes_in_universe | 14166 | 0.889696968 | 0.587198564 |
| RG_BR | Radial glia-like | BR | all_expressed_genes_minpct_0.01 | canonical_ISGs_only | 11 | 0.955518776 | 0.927272727 |
| RG_BR | Radial glia-like | BR | all_expressed_genes_minpct_0.01 | excluding_canonical_ISGs | 14155 | 0.863891618 | 0.586235442 |
| RG_BR | Radial glia-like | BR | all_expressed_genes_minpct_0.01 | non_ISG_genes | 14155 | 0.863891618 | 0.586235442 |
| RG_BR | Radial glia-like | BR | primary_exploratory_DE_tables | all_genes_in_universe | 191 | 0.983728031 | 0.969337283 |
| RG_BR | Radial glia-like | BR | primary_exploratory_DE_tables | canonical_ISGs_only | 10 | 0.915819639 | 0.903030303 |
| RG_BR | Radial glia-like | BR | primary_exploratory_DE_tables | excluding_canonical_ISGs | 181 | 0.984380504 | 0.964725882 |
| RG_BR | Radial glia-like | BR | primary_exploratory_DE_tables | non_ISG_genes | 181 | 0.984380504 | 0.964725882 |
| RG_FSS | Radial glia-like | FSS | all_expressed_genes_minpct_0.01 | all_genes_in_universe | 14176 | 0.899836318 | 0.613552483 |
| RG_FSS | Radial glia-like | FSS | all_expressed_genes_minpct_0.01 | canonical_ISGs_only | 11 | 0.972122258 | 0.927272727 |
| RG_FSS | Radial glia-like | FSS | all_expressed_genes_minpct_0.01 | excluding_canonical_ISGs | 14165 | 0.877363387 | 0.612651487 |
| RG_FSS | Radial glia-like | FSS | all_expressed_genes_minpct_0.01 | non_ISG_genes | 14165 | 0.877363387 | 0.612651487 |
| RG_FSS | Radial glia-like | FSS | primary_exploratory_DE_tables | all_genes_in_universe | 258 | 0.986839438 | 0.965453257 |
| RG_FSS | Radial glia-like | FSS | primary_exploratory_DE_tables | canonical_ISGs_only | 10 | 0.943701024 | 0.903030303 |
| RG_FSS | Radial glia-like | FSS | primary_exploratory_DE_tables | excluding_canonical_ISGs | 248 | 0.984444168 | 0.961385047 |
| RG_FSS | Radial glia-like | FSS | primary_exploratory_DE_tables | non_ISG_genes | 248 | 0.984444168 | 0.961385047 |

**Supplementary Table 2. Sample mapping and metadata annotation used in the analysis.**

| dataset | geo_accession | geo_sample_id | cell_system | condition | analysis_label | use_in_this_study |
| --- | --- | --- | --- | --- | --- | --- |
| Fetal neural scRNA-seq | GSE238140 | GSM7659279 | Fetal neural tissue | Mock | Mock | Primary fetal neural dataset |
| Fetal neural scRNA-seq | GSE238140 | GSM7659280 | Fetal neural tissue | ZIKV-BR | ZIKV_BR | Primary fetal neural dataset |
| Fetal neural scRNA-seq | GSE238140 | GSM7659281 | Fetal neural tissue | ZIKV-FSS/FSS13025 | ZIKV_FSS | Primary fetal neural dataset; Cambodian 2010 Asian-lineage FSS13025 isolate |
| Fetal neural scRNA-seq | GSE238140 | GSM7659282 | Fetal neural tissue | IFNÎ² | IFNb | Primary fetal neural dataset |
| Vero/moDC scRNA-seq | GSE230571 | GSM7226848_ZIKV-Vero-cells-1 | Vero cells | ZIKV-exposed | ZIKV_Vero_cells | Contextual comparison |
| Vero/moDC scRNA-seq | GSE230571 | GSM7226849_ZIKV-Vero-cells-2 | Vero cells | ZIKV-exposed | ZIKV_Vero_cells | Contextual comparison |
| Vero/moDC scRNA-seq | GSE230571 | GSM7226850_ZIKV-Vero-cells-3 | Vero cells | ZIKV-exposed | ZIKV_Vero_cells | Contextual comparison |
| Vero/moDC scRNA-seq | GSE230571 | GSM7226851_ZIKV-Vero-cells-4 | Vero cells | ZIKV-exposed | ZIKV_Vero_cells | Contextual comparison |
| Vero/moDC scRNA-seq | GSE230571 | GSM7226852_p22086-s001_1-5 | Primary human moDCs | moDC condition panel | moDC_panel | Contextual comparison |
| Vero/moDC scRNA-seq | GSE230571 | GSM7226854_p22086-s002_2-6 | Primary human moDCs | moDC condition panel | moDC_panel | Contextual comparison |
| Vero/moDC scRNA-seq | GSE230571 | GSM7226856_p22086-s003_3-7 | Primary human moDCs | moDC condition panel | moDC_panel | Contextual comparison |
| Vero/moDC scRNA-seq | GSE230571 | GSM7226858_p22086-s004_4-8 | Primary human moDCs | moDC condition panel | moDC_panel | Contextual comparison |
| Bulk RNA-seq | GSE97919 | GSM-level samples available in GEO metadata | Human neural samples | ZIKV-exposed/control | bulk_ZIKV/bulk_control | Contextual bulk RNA-seq analysis |

**Supplementary Table 3. Marker genes and curated ISG panel used in the analysis.**

| gene_list_category | cell_type_or_signature | genes | use_in_this_study | notes |
| --- | --- | --- | --- | --- |
| Cell-type marker | Progenitor-enriched radial glia-like | SOX2, PAX6, NES | Cell-type annotation | Interpreted cautiously as progenitor-enriched radial glia-like because SOX2, PAX6, and NES are shared across progenitor states |
| Cell-type marker | Cycling NPC | MKI67, TOP2A, HMGB2 | Cell-type annotation | Cycling progenitor-associated markers |
| Cell-type marker | Excitatory neuron | SLC17A7, NEUROD6 | Cell-type annotation | Excitatory neuron-associated markers |
| Cell-type marker | Inhibitory neuron | GAD1, GAD2, DLX1 | Cell-type annotation | Inhibitory neuron-associated markers |
| Cell-type marker | Astrocyte | GFAP, AQP4 | Cell-type annotation | Astrocyte-associated markers |
| Cell-type marker | Microglia | C1QA, C1QB, TYROBP | Cell-type annotation | Microglia-associated markers |
| Cell-type marker | OPC | PDGFRA, OLIG1, OLIG2 | Cell-type annotation | Oligodendrocyte precursor-associated markers |
| Cell-type marker | Endothelial | PECAM1, VWF | Cell-type annotation | Endothelial-associated markers |
| ISG signature | Canonical ISG panel | IFITM1, MX1, OAS1, OAS2, OAS3, IFIT1, IFI6, ISG15, STAT1, IRF7, RSAD2 | ISG heatmaps, ISG summary plots, AddModuleScore, and ZIKV-IFNÎ² correlation sensitivity analyses | Curated canonical interferon-stimulated gene panel |
